# The Role of Imaging Modalities in Diagnosing Dysphagia: A Clinical Review

**DOI:** 10.7759/cureus.16786

**Published:** 2021-07-31

**Authors:** Haider Ghazanfar, Elona Shehi, Jasbir Makker, Harish Patel

**Affiliations:** 1 Internal Medicine, Bronxcare Health System, Bronx, USA; 2 Medicine/Gastroenterology, Bronxcare Health System, Bronx, USA; 3 Gastroenterology, Bronxcare Hospital Center, Bronx, USA; 4 Internal Medicine, Bronxcare Hospital Center, Bronx, USA

**Keywords:** dysphagia, elderly, videofluoroscopic swallow study, fiberoptic endoscopic evaluation of swallowing, high-resolution pharyngeal manometry, high resolution esophageal manometry, barium swallow, esophagogastroduodenoscopy

## Abstract

Dysphagia, which is characterized by difficulty in oro-gastric bolus transit, is a common condition. It is broadly classified into oropharyngeal or esophageal pathology. A wide array of differentials for dysphagia and initial clinical suspicion of oropharyngeal or esophagus etiology can assist in further evaluation. Fiberoptic endoscopic evaluation of swallowing (FEES) and videofluoroscopic swallow study (VFSS) are the preferred modalities for assessing oropharyngeal bolus transit, residual, as well as determining the risk of laryngeal aspiration. High-resolution pharyngeal manometry (HRPM) is an emerging modality for optimal topographical and pressure assessment of pharyngeal anatomy. HRPM provides improved assistance in evaluating the strength of the pharyngeal muscular contraction. Esophagogastroduodenoscopy (EGD) is the preferred exam for patients with suspected esophageal etiology of dysphagia. Barium swallow provides luminal assessment and assists in evaluating esophageal motility; it is non-invasive, but therapeutic interventions like biopsy cannot be performed. High-resolution esophageal manometry (HREM) has added another dimension in the diagnosis of esophageal motility disorders. The purpose of this review article is to help internists and primary care providers get a better understanding of the role of various imaging modalities in diagnosing dysphagia in the elderly population. This article also provides a comprehensive review and detailed comparison of these imaging modalities based on the latest evidence.

## Introduction and background

Dysphagia is defined as an inability to swallow or a difficulty in the passage of food from the mouth to the stomach due to functional or mechanical obstruction of the luminal organ, including the oropharynx, esophagus, or gastric cardiac. According to the 2012 National Health Interview Survey, approximately nine million adults have reported a swallowing problem [1]. The higher incidence of dysphagia in the elderly is well documented; however, there is scarce data on its precise etiology and diagnostic workup. Dysphagia is commonly managed by specialists, including gastroenterologists, otolaryngologists, and speech-language pathologists. This article focuses on the most common questions encountered while managing the oropharyngeal and esophageal stages of dysphagia in the elderly. The anticipatory stage of dysphagia is not discussed in the article.

The purpose of this review article is to help internists and primary care providers get a better understanding of the role of various imaging modalities in diagnosing dysphagia in the elderly population. It also provides a comprehensive review and detailed comparison of these imaging modalities based on the latest evidence.

What age group defines the elderly?

The population of the world is aging rapidly. Currently, there is no universally accepted definition for the term "elderly." Age of more than 65 years has been accepted in most places as the chronological definition of the word "elderly" [2]. With increasing life expectancy and decreasing fertility rate, the proportion of the elderly among the total population of the world is expected to increase further. According to the United States Census Bureau, adults aged more than 65 years account for 23% of the total population.

How common is dysphagia in the elderly?

The prevalence of dysphagia is higher in the elderly population as compared to the general population. The prevalence increases as the functional capacity of the patients decline. The prevalence of dysphagia is approximately 30-40% in elderly patients living independently, compared to 52.7-69.6% in elderly patients residing in nursing homes [3-5]. According to a multicenter study done in nursing homes in Spain, female gender (p = 0.06) and Barthel Index score (p = 0.02) were significantly associated with oropharyngeal dysphagia [3]. About 43% of the elderly patients with aspiration pneumonia had oropharyngeal dysphagia [3]. A study conducted among institutionalized elderly individuals in Taiwan showed that 64.2% of patients in skilled nursing facilities and 44.6% of patients in intermediate-care facilities suffered from swallowing impairments [4]. According to a study done on nursing home residents in South Korea, feeding time of more than 20 minutes, meal type, functional status, and BMI of less than 20 were associated with dysphagia [5]. The prevalence of dysphagia is expected to increase as the world’s population ages further. The actual prevalence is assumed to be much more than the reported prevalence because most of the elderly consider dysphagia symptoms as part of the normal aging process. The elderly population is at considerable risk of dysphagia due to the normal aging process and susceptibility to various diseases.

## Review

The cause of dysphagia in the elderly: is it oropharyngeal or esophageal? Which type of dysphagia is more common: oropharyngeal or esophageal?

"Geriatric syndrome" consists of clinical conditions highly prevalent in the elderly population but do not fit into any specific disease category [6]. For a clinical condition to be considered a geriatric syndrome, it must have a significant clinical impact on the patient's quality of life. Dysphagia has been shown to have a detrimental effect on the quality of life of the elderly.

Dysphagia can be anatomically divided into oropharyngeal dysphagia and esophageal dysphagia. Patients with oropharyngeal dysphagia experience difficulty in the movement of food from the mouth into the pharynx and the esophagus while patients with esophageal dysphagia have difficulty in the passing of the food through the esophagus. The initial step in dysphagia management is to classify it on the basis of its pathogenesis into the oropharyngeal or the esophageal [7]. The evaluation and the management differ for both entities, and hence it is imperative to distinguish the etiologies.

Based on the review of the current literature, oropharyngeal dysphagia is more common in the elderly. Various studies have suggested that oropharyngeal dysphagia should be considered as a geriatric syndrome [8].

Oropharyngeal dysphagia

Oropharyngeal dysphagia is more prevalent in the elderly population as compared to esophageal dysphagia [9]. The elderly population is at risk of developing dysphagia due to the normal aging process, their frail state, and risk factors for illnesses that affect the normal swallowing mechanism. Studies have shown that approximately 25-50% of the patients with stroke have dysphagia [10,11]. According to a study conducted among elderly patients in Spain, about 68.4% of the patient with oropharyngeal dysphagia had dementia [3]. Dysphagia occurs in 50% and 31.3% of the patients with Parkinson’s disease and multiple sclerosis, respectively [12]. Etiologies of oropharyngeal dysphagia are listed in Table 1 [3,10-12].

**Table 1:** Etiologies of oropharyngeal dysphagia

Etiologies
Motor Disorder
Central and Peripheral Nervous System
Stroke
Head Trauma
Primary Brain Malignancies or Metastasis to the Brain
Metabolic Encephalopathy
Neurodegenerative Disorder
Alzheimer's
Multiple Sclerosis
Amyotrophic Lateral Sclerosis
Parkinson's Disease
Huntington's Disease
Wilson's Disease
Poliomyelitis
Neuromuscular
Myasthenia Gravis
Thyroid Myopathy
Muscular Dystrophies
Polymyositis and Dermatomyositis
Structure Disorder
Head and Neck Malignancy
Head and Neck Surgery
Radiation Injury
Extrinsic Compression (Goiter, Malignancy, Cervical Osteophytes and Skeletal Abnormalities)
Lateral Pharyngeal Pouch or Diverticula
Plummer-Vinson (Proximal Esophageal Web)
Zenker Diverticulum
Cricopharyngeal Achalasia
Poor Dentition
Sjogren's Syndrome

Esophageal dysphagia

The etiologies of esophageal dysphagia can be divided into structural and motor disorders. The structural disease can be further subdivided into intrinsic and extrinsic causes, while the motor disorder can be further subdivided into primary and secondary motility disorders. Esophagitis secondary to gastroesophageal reflux is one of the most common causes of dysphagia among the elderly population. Elderly patients are also at risk of developing dysphagia secondary to foreign bodies because of chewing problems, dentures, and the effect of aging on the swallowing mechanisms. According to a study done in China, dental prostheses and food bolus account for approximately 47.5% of foreign bodies in the esophagus in the elderly population [13]. The incidence of achalasia peaks around the third and sixth decades of life. Studies have shown that the incidence of achalasia increases with age [14].

The differentiation of achalasia from pseudoachalasia is extremely important in the elderly population. Pseudoachalasia in the elderly can result from malignancy and paraneoplastic syndrome [15]. Etiologies of esophageal dysphagia are listed in Table 2 [13,15].

**Table 2:** Etiologies of esophageal dysphagia CREST: calcinosis, Raynaud phenomenon, esophageal dysmotility, sclerodactyly, and telangiectasia

Etiologies
Structural Disorders
Intrinsic Disease
Esophagitis; Infectious (Candida, Herpes Simplex Virus, Cytomegalovirus), Pill-induced Esophagitis, Gastric Reflux-induced
Mucosal Rings and Webs: Schatzki Ring, Plummer-Vinson, and Eosinophilic Esophagitis
Strictures: Anastomotic, Caustic Ingestion, Peptic, Radiation-induced
Systemic Disease: Dermatology Disease (Bullous Pemphigoid, Lichen Planus, Pemphigus Vulgaris, Steven-Johnson Syndrome), Gastroenterology Disease (Crohn's Disease), Connective Tissue Disorder (Scleroderma)
Foreign Body
Primary or Metastatic Malignancy
Extrinsic Compression
Cardiomegaly (Enlarged Left Atrium)
Mediastinal Mass (Lung Cancer, Lymphoma, Enlarged Lymph Node)
Spinal Osteophytes
Vascular Compressions (Dysphagia Aortica, Dysphagia Lusoria)
Motor Disorders
Primary Motility Disorder
Achalasia
Diffuse Esophageal Spasm
Hypertensive Lower Esophageal Sphincter
Ineffective Esophageal Motility Disorder
Nutcracker Esophagus
Secondary Motility Disorder
Amyloidosis
Chagas Disease
Connective Tissues Disorder
CREST Syndrome
Diabetes
Medications
Paraneoplastic Syndrome
Scleroderma
Thyroid Dysfunction

General clinical assessment to differentiate between oropharyngeal versus esophageal dysphagia

Clinical assessment does assist in differentiating oropharyngeal from esophageal etiologies. Underlying neuromuscular disease in a patient is highly suggestive of oropharyngeal etiologies. However, there is a lack of validated questionnaires to assess the specificity and sensitivity of clinical assessment in differentiating oropharyngeal from esophageal etiologies. Based on the timing of the initiation of the difficulty in swallowing, associated symptoms, comorbid clinical condition, and the relief of the symptoms, some of the authors have suggested the use of a clinical questionnaire to help differentiate between the two etiologies [16].

Patients with oropharyngeal dysphagia usually present with difficulty in initiating swallowing, as well as choking, coughing, drooling, and nasal regurgitation. Most of the patients with oropharyngeal dysphagia experience associated weight loss and a history of recurrent aspiration pneumonia. Oral and pharyngeal dysfunction in these patients can lead to dysarthria and dysphonia, respectively. Patients with esophageal dysphagia usually present with the sensation of food getting stuck in their throat or chest.

One of the critical differences between oropharyngeal and esophageal dysphagia is the onset of the symptoms. Patients with oropharyngeal dysphagia usually start having symptoms right after swallowing, while patients with esophageal dysphagia start developing symptoms after several seconds after eating. 

Patients presenting with dysphagia need to be approached systemically so that the underlying etiology can be identified.

Imaging and endoscopic imaging studies for the evaluation of dysphagia

A detailed history and physical examination play a pertinent role in identifying the etiology of dysphagia. As per our literature review, there is currently no clinical bedside evaluation test designed specifically to identify which imaging modality to use in diagnosing dysphagia in the elderly [17]. We have focused more on the imaging modalities used in diagnosing oropharyngeal dysphagia, as it is the most common type of dysphagia in the elderly. Invasiveness of the imaging modality and the need to use anesthesia are the two critical factors in deciding which imaging modality to use. The patient's ability to participate in the test also plays an essential role in deciding which imaging modality to opt for.

 Imaging modalities that are useful in helping diagnose oropharyngeal dysphagia include:

· Videofluoroscopic swallow study (VFSS) or modified barium swallow

· Fiberoptic endoscopic evaluation of swallowing (FEES)

· Pharyngeal manometry

· Barium swallow

Imaging modalities found to help diagnose esophageal dysphagia include:

· Barium swallow

· Manometry

· Esophagogastroduodenoscopy (EGD)

Modalities for evaluating oropharyngeal dysphagia

Fiberoptic Endoscopic Evaluation of Swallowing (FEES)

FEES is helpful in the diagnosis of oropharyngeal dysphagia and in assessing the safety and efficacy of the swallowing mechanisms. This procedure involves passing a flexible endoscopic instrument through the nose into the throat. It can help identify if there is any obstruction due to any structural lesions or mass. It is also helpful in assessing the risk of aspiration in patients with dysphagia.

FEES examination protocol includes anatomic-physiological assessment and the swallow evaluation [18]. Velopharyngeal closure and the appearance of the larynx and hypopharynx are assessed and tested. The secretion and frequency of the dry swallow pharyngeal wall medialization and larynx functions are assessed in the anatomic-physiological assessment. Swallow evaluation includes the assessment of food ingestion with different consistency and volume along with the presence of the cough reflex if there is a laryngeal swallow.

FEES is a bedside procedure and preferred for patients for whom transportation and positioning for the modified barium swallow is an issue. FEES provides an anatomical and mucosal assessment of the oral pathway. It is preferred for patients in whom biofeedback is recommended for the management of dysphagia.

According to a study performed in acute stroke patients, FEES helped in modifying the diet in 69.1% of patients with acute stroke [19]. Another study done in a multidisciplinary outpatient clinic showed that FEES could help detect oropharyngeal dysphagia in patients with myotonic dystrophy [20]. A retrospective review done in a tertiary care center concluded that FEES plays a vital role in modifying the diet in patients with neurodegenerative disorders and can help prevent complications associated with aspiration [21]. In the same study, 88% of the patients were advised about a functional change in management after undergoing FEES. Patients with aspiration and penetration on FEES were found to have a higher mean Eating Assessment Tool-10 (EAT-10) score than a patient with a normal FEES result. A study done in patients with acute traumatic brain injury concluded that FEES is a sensitive diagnostic tool for the assessment pharyngeal stage of dysphagia. In the study, around 36% of the patients were diagnosed with dysphagia associated with aspiration [22]. Location of pooling, liquid bolus consistency, and post-swallow pharyngeal pooling identified on FEES has been found to impact the probability of aspiration in head and neck cancer patients presenting with dysphagia [23]. According to a systemic review and meta-analysis, FEES had a sensitivity of 88%, 97%, and 97% for aspiration, penetration, and laryngopharyngeal residues, respectively [24]. In the same study, it was concluded that FEES has greater sensitivity in all the components as compared to VFSS (p: <0.05) [25]. FEES has been found helpful in diagnosing dysphagia in patients with amyotrophic lateral sclerosis [21], acute traumatic brain injury [22], acute stroke [19], head and neck cancer [23], and muscular dystrophies [20].

Videofluoroscopic Swallow Study (VFSS)

VFSS, also known as modified barium swallowing examination (MBS), is another imaging modality that helps diagnose oropharyngeal dysphagia. It is considered to be the gold standard for the diagnosis of oropharyngeal dysphagia. In this procedure, the patient is asked to swallow liquid or solid barium. Fluoroscopy is then used to get a real-time image as the patient swallows. It also helps detect the presence of aspiration and assists in identifying the underlying etiology of aspiration [25].

VFSS requires patient co-operation for proper positioning. It is a less invasive procedure as compared to FESS. It assists in the proper identification of the upper esophageal stricture or hypertonicity. VFSS can diagnose the fistula in the postoperative evaluation of dysphagia [18].

VFSS has also been shown to be helpful in diagnosing cervical dysphagia. According to a study, VFSS was able to detect esophageal alteration in 31% of the patients without any prior established etiological diagnosis [26]. VFSS has also been indicated in asymptomatic patients in whom aspiration is suspected [27]. VFSS has been shown to help in determining an appropriate diet in patients with acute stroke. A study done in acute stroke patients concluded that VFSS helped in modifying the diet in 80% of the patients and helped reduce the incidence of aspiration pneumonia [28]. According to a systemic review and meta-analysis, VFSS has a sensitivity of 77%, 83%, and 80% for aspiration, penetration, and laryngopharyngeal residues, respectively [21]. VFSS has been shown to help diagnose dysphagia in patients with acute stroke, traumatic brain injury, Parkinson's disease, amyotrophic lateral sclerosis, multiple sclerosis, and Alzheimer’s disease [27]. Pharyngeal barium residue after initial swallow in VFSS is useful in assessing the severity of dysphagia in patients with spinal and bulbar muscular atrophy [29]. VFSS cannot be done in patients who are unable to follow commands and collaborate during the procedure. In these patients, FEES is preferred.

High-Resolution Pharyngneal Manometry (HRPM)

HRPM is an emerging modality utilized to understand the precise mechanism of oropharyngeal dysphagia and identifying those at risk for dysphagia in a neurological disorder like Parkison’s disease.

The High-Resolution Pharyngeal Manometry International Working Group first defined the protocol and the metrics for HRPM [30]. The HRPM parameters are categorized into three classes: pharyngeal lumen occlusive pressures, hypopharyngeal intrabolus pressures, and upper esophageal sphincter (UES) function. At the Delphine convention, a consensus was reached to implement eight of the metrics in performing HRPM.

One of the advantages of HRPM is that it can be performed without ingesting any barium bolus. HRPM is safe in evaluating dysphagia in patients at high risk of aspiration [31]. Patients with severe Parkinson's disease have been shown to have decreased oropharyngeal and velopharyngeal pressures [31]. Studies have shown that patients with dysphagia were found to have lower pressure throughout the pharynx and UES [30]. High-resolution manometry (HRM) effectively detects changes in swallowing-related pressures in patients with early Parkinson's disease even before the onset of dysphagia's signs and symptoms [32]. Patients with dysphagia have been shown to have weaker mesopharyngeal and hypopharyngeal contractility on HRM [33].

Restriction of the UES to bolus flow can be assessed by measuring hypopharyngeal intrabolus pressure. Studies have shown that hypopharyngeal intrabolus pressure is higher in patients with dysphagia [30]. One of the other advantages of HRPM is its ability to measure UES relaxation pressure and time intervals. Higher UES relaxation pressures are associated with a shorter relaxation time (r = -0.500, p: <0.050) in a study done on stroke patients [33].

Patients with dysphagia associated with aspiration have been found to have a shorter UES relaxation time interval as compared to patients without aspiration (p: <0.05) [33]. Incomplete relaxation of UES has been associated with Zenker's diverticulum [34]. Low resting pressures of the UES are seen in dysphagia patients with myasthenia gravis, myotonic dystrophy, and amyotrophic lateral sclerosis [35].

Modalities for the evaluation of esophageal dysphagia

A detailed esophageal luminal, mucosal, and motility assessment is required in patients with suspicion of esophageal dysphagia. EGD is usually preferred due to mucosal and luminal evaluation with the added benefit of performing an esophageal biopsy if needed; however, it is invasive and often requires sedation. Barium swallow or esophagogram is a viable alternative but often requires subsequent EGD to confirm the diagnosis.

Barium Swallow

A barium swallow is an imaging modality that uses real-time fluoroscopy and barium to evaluate for esophageal dysphagia. It is helpful in assessing for any morphologic and motility abnormalities in the pharynx and esophagus. Over the years, several advancements have been made in the technique of the barium swallow.

Barium swallow has been shown to be useful in diagnosing esophageal webs, rings, and diverticula [36]. Schatzki rings with a diameter of less than 13 mm are usually associated with dysphagia [37]. According to a study, Schatzki ring accounts for 10.1% of patients with esophageal dysphagia [38].

Barium swallow has been shown to help diagnose reflux, infectious, caustic, drug, and radiation-induced esophagitis [36]. In the double-contrast barium swallow study, the patient has to swallow an effervescent agent and high-density barium. One of the advantages of double-contrast examination is that this technique provides superior mucosal detail compared to single-contrast barium swallows. It has been shown to have a sensitivity of 35% and specificity of 79% in diagnosing reflux esophagitis [39]. One of the reasons for the low sensitivity of double-contrast barium in diagnosing reflux esophagitis is the use of proton pump inhibitors before the test.

Esophageal tumors appear as intraluminal or intramural filling defects on the barium swallow. Some studies have also reported that double-contrast barium swallow sensitivity in diagnosing esophageal cancers is more than 95% [40]. According to a study, none of the patients diagnosed as having benign structures on barium swallow was found to have a malignant tumor on endoscopy [41]. The same study showed that 100% of the patients identified as having malignant stricture were found to have malignant tumors on endoscopy [41]. A barium swallow is also helpful in identifying extrinsic compression of the esophagus.

A double-contrast barium swallow is also useful in assessing esophageal motility. "Bird's beak" and "megaesophagus" appearance on barium swallow is associated with primary achalasia [42]. Narrowing on barium swallow extending more than 3.5 cm above the gastroesophageal junction, which appears as a "rat-tail sign," is commonly associated with secondary achalasia [43]. "Corkscrew" or "rosary beard" appearance on barium swallow is characteristically associated with diffuse esophageal spasm [44].

Barium esophagogram is rarely used as a stand-alone investigation, except in few cases such as follow-up investigation for achalasia management assessment. Timed barium swallow denotes the comparison of the esophageal barium column's height at the start and after five minutes. Partial emptying of the barium is suggestive of achalasia [45]. The 50% reduction in the barium column at five minutes after the treatment for achalasia is considered a sign that the treatment was successful [46].

A barium swallow can be done before endoscopy to assess the risk of perforation and aspiration. A barium swallow is contraindicated in patients with esophageal perforation or pregnancy. Abdominal pain and constipation are the two common complications associated with a barium swallow. Aspiration of barium can lead to hypersensitivity pneumonitis, while barium leakage through perforation can result in mediastinitis and peritonitis.

Esophagogastroduodenoscopy (EGD)

EGD is the most essential diagnostic and therapeutic modality in the management of dysphagia. EGD may conclude the diagnosis in patients with unrevealing imaging studies, like barium swallow. Therapeutic intervention, including stricture dilation and esophageal biopsy, can be performed during EGD. Esophageal mass requires a biopsy to establish a diagnosis. However, even in patients with a normal-appearing esophagus, biopsies in the middle and lower esophagus are recommended to evaluate for eosinophilic esophagitis (EoE) [47]. In high-risk individuals, lower esophageal assessment for the esophagitis and Barrett’s esophagus can be performed. Dysphagia due to gastric cardiac pathologies is often missed with other imaging modalities, and it can be diagnosed with retro-flexed view evaluation during an EGD. 

Clinical Outcomes Research Initiative (CORI) is a national database that was established in 1995 to assess the use and outcome of gastrointestinal (GI) endoscopic procedures in diverse clinical settings. Data files from these practice centers are sent to the National Endoscopy Database (NED) after patient and physician identifiers are removed. This data is then used for research purposes. A review of the CORI database revealed that a total of 30,377 EGDs were performed for dysphagia evaluation over a study period of six years. The study analyzed the data until 2006 and reported esophageal stricture to be the most common etiology of dysphagia, accounting for 40% of procedures performed for dysphagia [48]. The elderly (those above 60 years of age) are more likely to have esophagus strictures than those below 60 years of age. Another retrospective analysis showed that EGD was able to identify major pathologies and abnormal findings in 54% and 70% of the patients with dysphagia, respectively [49]. The study showed that EGD was most likely to diagnose dysphagia's underlying pathology in male patients and patients with heartburn and odynophagia [49]. According to another study, EGD was found to have a predictive value of 76.2% [50].

Endoscopy helps to diagnose esophageal structural abnormalities such as mucosal abnormalities, rings, retained food, strictures, and masses. Esophageal strictures and gastroesophageal reflux disease (GERD) are the most common EGD findings [50,48]. A biopsy can be done during endoscopy to rule out underlying malignancy. Studies have shown that multiple mucosal biopsies have a sensitivity of 96% in diagnosing esophageal cancer [51]. Complications associated with EGD include infection, bleeding, and esophageal perforation.

High-Resolution Esophageal Manometry (HREM)

Manometry is a valuable imaging modality useful in diagnosing esophageal dysphagia and is particularly helpful in patients in whom a motility disorder is suspected. HREM is more sensitive than conventional manometry. The absence of peristalsis and incomplete relaxation of the lower esophageal sphincter (LES) are the key features of achalasia in conventional manometry [52].

The absence of peristalsis and the elevated integrated relaxation pressure (IRP) is the hallmark of achalasia on HREM. IRP value of more than 15 mmHg is indicative of achalasia as per the Chicago Classification Criteria [53]. An IRP value is consistent with the severity of achalasia [54]. Achalasia can be classified into three types by HREM. Type 1 includes patients with 100% failure of peristalsis, type 2 includes patients with absent peristalsis and ≥20% swallowing with panesophageal pressurization, and type 3 includes patients with absent peristalsis and ≥20% swallowing with preserved spastic contractions [53]. Studies have shown that type 2 achalasia is more amenable to treatment therapies than type 1 and type 3 [55]. Treatment options available for type 2 achalasia include botulinum toxin, pneumatic dilation, and Heller myotomy. HREM has been shown to have a sensitivity of 98% and specificity of 96% in diagnosing achalasia [56].

Diffuse esophageal spasm (DES) is defined by normal LES relaxation (IRP ≤15 mmHg) and a premature contraction in at least 20% of water swallows [56]. The hypercontractile esophagus is defined as the occurrence of ≥20% of swallows with a distal contractile integral (DCI) of more than 8,000 mmHg•s•cm and a normal latency [57]. Weak, ineffective pressure waves and low to absent basal LES pressure are the typical findings seen in patients with scleroderma and other connective tissue diseases [58,59].

An integral approach to dysphagia

Given the wide complexity of underlying etiology and overlapping mechanisms, it may be challenging to have a rationalized algorithm for ordering a specific workup for dysphagia. The clinical acumen classifies the pathogenesis into oropharyngeal or esophageal in the majority of patients [60]. It is prudent to understand each diagnostic modality's mechanism, pros, and cons in a given clinical scenario for the optimal management of dysphagia. We have summarized the key features of each diagnostic modality in Table 3.

**Table 3:** Comparison of various diagnostic imaging modalities for dysphagia

Procedure	Indication	Pros	Cons	Disease
Fiberoptic endoscopic evaluation of swallowing (FEES)	Patients with suspicion of aspiration/larynx penetration	Assesses pharyngeal stage before, during, and after the swallow	Whiteout period	Diagnosing oropharyngeal dysphagia due to neurodegenerative disorder and neuromuscular disorder
		Portable	Cannot assess the oral phase and cervical esophageal phase of swallow	Diagnosing oropharyngeal dysphagia due to structural disorder
		Use of real food (no barium)	Trace aspiration may be missed	
		No radiation exposure	Difficult to perform in patients with craniofacial trauma, severe dementia, brain trauma, and confused or comatose patients	
		Can assess multiple swallows of different consistencies	Nasoendoscopy discomfort	
		Fatigue examination is possible due to the longer duration of the exam	Time-consuming disinfection process	
		Less expensive		
		Directly visualizes laryngeal and pharyngeal surface		
		Assessment of velopharyngeal closure		
		Therapy biofeedback		
		Can be performed in the outpatient setting		
		Three-dimensional view		
		Pooling management view		
Videofluoroscopic swallow study (VFSS)	Patients with suspicion of problem in the oral phase of swallowing	Assesses oral, pharyngeal, and cervical esophageal phases of swallow before, during, and after the swallow	Lower sensitivity for microaspiration	Diagnosing oropharyngeal dysphagia due to stroke, head trauma, primary brain malignancies, or metastasis to the brain
	Patients with suspicion of aspiration/larynx penetration	Detects aspiration	Radiation exposure	Diagnosing oropharyngeal dysphagia due to neurodegenerative disorder and neuromuscular disorder
	Patient with complaints of food getting stuck in the throat		Limited duration of the examination	
			Not portable	
			Difficult to perform in patients who are unable to leave the room, ventilator-dependent patients, patients in intensive care, and uncooperative patients	
			Expensive procedures	
			Unable to view laryngeal anatomy	
			Unable to do fatigue evaluation	
			Underestimation of pooling matter	
			Aspiration of barium can lead to hypersensitivity pneumonitis	
High-resolution pharyngeal manometry (HRPM)	Patient with suspected pharyngeal motility disorder	Does not require ingestion of barium bolus	No direct visualization of pharyngeal and laryngeal structure	Oropharyngeal dysphagia due to Parkinson's disease, myasthenia gravis, myotonic dystrophy, and amyotrophic lateral sclerosis
		Assesses mesopharyngeal and hypopharyngeal contractility	Unable to assess the presence of aspiration	Oropharyngeal dysphagia due to Zenker diverticulum
		Assesses hypopharyngeal intrabolus pressure	Unable to assess the degree of pharyngeal residue	
		Assesses upper esophageal sphincter relaxation pressure and time interval		
Barium swallow	Patient with suspected esophageal dysphagia	Provides good anatomical detail of the esophagus	Radiation exposure	Esophageal dysphagia due to esophageal webs, rings, and diverticula
		Can identify extrinsic compression of the esophagus	Difficult to perform in patients who are unable to leave the room, ventilator-dependent patients, patients in intensive care, and uncooperative patients	Esophageal dysphagia due to reflux, infectious, caustic, drug, and radiation-induced esophagitis
		Widely available	Cannot detect dynamic disorders	Esophageal dysphagia due to extrinsic compression of the esophagus
			Cannot detect the pharyngeal cause	Esophageal dysphagia due to primary and secondary achalasia
			Cannot be performed in patients with bowel obstruction, suspected perforation, and postoperative assessment for leak	
			Aspiration of barium can lead to hypersensitivity pneumonitis while barium leakage through perforation can lead to mediastinitis and peritonitis	Esophageal dysphagia due to diffuse esophageal spasm
Esophageal manometry	Patient with suspected primary and secondary motility disorder	Assesses for the absence of peristalsis	No direct visualization of the esophageal structure	Esophageal dysphagia due to primary and secondary achalasia
		Assesses lower esophageal integrated relaxation pressure	Inability to record esophageal motility function for an extended time interval in the case of standard high-resolution manometry	Esophageal dysphagia due to diffuse esophageal spasm
Esophagogastroduodenoscopy (EGD)	Patients with suspected esophageal dysphagia	Assesses esophageal structural abnormalities	Risk of infection, bleeding, and perforation	Diagnosing esophageal dysphagia due to esophageal structural abnormalities such as mucosal abnormalities, rings, retained food, strictures, and mass
		Biopsy can be done during the procedure and the sample can be sent for histopathology examination	Unable to diagnose motility disorder	
		Therapeutic modality	Invasive procedure	
			Complications due to sedation	

## Conclusions

Dysphagia is a common ailment in the elderly. FEES and VFSS have been utilized to diagnose and manage oropharyngeal dysphagia, and newer modalities like HRPM have also emerged. EGD and barium swallow are preferred for diagnosing esophageal dysphagia's luminal component, and HREM can help in identifying esophageal motility disorder.
